# Diagnostic Yield of Corneal, Contact Lens, and Lens Fluid Cultures in Microbial Keratitis: A 12-Year Single-Center Study

**DOI:** 10.1097/ICO.0000000000004045

**Published:** 2025-12-11

**Authors:** Matteo Airaldi, Alfredo Borgia, Davide Romano, Siddarth Nardeosingh, Tobi Somerville, Vito Romano, Timothy Neal, Stephen B. Kaye

**Affiliations:** * Liverpool University Hospitals NHS Foundation Trust, Liverpool, United Kingdom;; † Department of Eye and Vision Science, University of Liverpool, Liverpool, United Kingdom;; ‡ Department of Molecular and Translational Medicine, University of Brescia, Brescia, Italy;; § Cornea Unit, “Mons. Dimiccoli” Teaching Hospital, Barletta, Italy;; ¶ Department of Medical and Surgical Specialties, Radiological Sciences, and Public Health, Ophthalmology Clinic, University of Brescia, Brescia, Italy; and; ║ Department of Infection and Immunity, Liverpool University Hospitals NHS Foundation Trust, Liverpool, United Kingdom.

**Keywords:** microbial keratitis, contact lens-related keratitis, bacterial keratitis, contact lens fluid

## Abstract

**Purpose:**

To determine the yield of culturing contact lenses (CL) and contact lens fluid/cases (CLF) to isolate recognized pathogens in suspected microbial keratitis (MK).

**Methods:**

Data from 4298 ocular samples of MK collected at the Royal Liverpool University Hospital between January 2012 and December 2023 were reviewed. The isolation rates and proportion of recognized pathogens were compared between corneal impression membranes (CIM) and scrapes (CS), CL, and CLF. Chi-square tests and mixed-effects logistic models were used to assess differences in distribution among all isolates and subgroups of samples.

**Results:**

The overall isolation rate was 54.1%, with CL having the highest rate ([348/466], 74.7%), followed by CIM ([1822/2940], 62%), CS ([126/566], 22.3%), and CLF ([23/317], 7.3%). CLF, however, had the highest proportion of recognized pathogens ([22/28], 78.6%), followed by CL ([269/531], 50.7%), CS ([71/142], 50%), and CIM ([728/2218], 32.8%). CIM and CS predominantly detected Gram-positive bacteria, whereas CLF had the highest rates of Acanthamoeba ([13/28], 46.4%) and fungal isolates ([4/28], 14.3%). CL and/or CLF cultures led to altered treatment in 10.5% of cases.

**Conclusions:**

In suspected MK, culturing the CLF and CL can increase the probability of detecting a recognized pathogen that in turn may help guide treatment.

## Abstract 2

Supplemental Digital Content is Available in the Text.

Contact lens (CL) wear is a recognized risk factor for microbial keratitis (MK).^1,2^ They are worn by an estimated 140 million individuals worldwide, with an ever expanding user base.^1,2^ The increase in CL wear has been accompanied by a rise in CL-related infections, including an estimated 71,000 annual cases of CL-related MK.^2,3^

Microbial adherence to CLs is promoted by the formation of biofilms, present despite good hygiene compliance.^4^ Common contaminants of CLs and CL cases include *Pseudomonas*, *Staphylococcal*, and *Streptococcal* taxa.^4,5^ Although a multitude of microorganisms may contaminate CLs, Gram-negative (G-ve) rods are more prevalent in CL-related MK, whereas endogenous sources of infection are more common in non–CL-related MK.^6^ Regional differences seem to affect less the variability of pathogens involved in CL-related MK; however, climatic conditions can play a role in disease severity and causative organisms, with an increased relevance of fungal contamination in warmer regions of the world.^7–9^ CL use is also one of the main risk factors for *Acanthamoeba* keratitis, which although rare can have devastating outcomes.^9,10^

Case series have reported that CL culture may help to detect presumed causative microorganisms in CL-related MK, especially where corneal cultures are negative.^6,11,12^ The difficulty, however, in assessing the value of culturing CLs and CLFs is that previous studies have not clearly distinguished between pathogens and microorganisms that are potential contaminants or of uncertain pathogenic role. The aim of this study, therefore, was to determine the yield of CL and CLF for the isolation of recognized pathogens in suspected MK.

## MATERIALS AND METHODS

### Study Design and Setting

This retrospective study included corneal and contact lens samples collected at the Royal Liverpool University Hospital (RLUH) between January 2012 and December 2023. The study received approval from the Ethics Committee of the RLUH (reference number: *Ophth/SE/2024–25/10*) and adhered to the principles outlined in the Declaration of Helsinki.

### Data Sources and Study Groups

Microbiological data of ocular samples collected for culture and sensitivity in the study period were extracted from laboratory records. Variables collected through microbiology records included a unique patient identifier, date of sampling, type of sample, and a unique microorganism code for each of the isolates that originated from the sample. Data extraction was filtered to include only CL, CLF, corneal impression membrane (CIM), and cornea scrape (CS) samples. The isolates’ stain (G +ve bacteria, G −ve bacteria, Acanthamoeba, fungi) and their relative pathogenicity (pathogen vs. of unknown clinical significance) was then matched to each unique microorganism code according to previously published works.^13^

Organisms well established as causative agents of MK—such as *Pseudomonas aeruginosa*, *Staphylococcus aureus*, *Streptococcus pneumoniae*, *Serratia* spp., *Acanthamoeba* spp., and fungal species including *Fusarium* and *Aspergillus*—are known to express a range of virulence factors that enhance their pathogenic potential in corneal infection and were therefore classified as pathogenic in this study.^13^ Other organisms in addition to the ones above were classified as pathogenic when they have not been reported as part of the normal ocular surface microbiome in previous studies^14^ and have been implicated in MK in more than single case reports. This included other G-ve organisms (*Citrobacter*^15,16^*, Enterobacter*,^17^ *Escherichia coli*,^18^ *Haemophilus*,^19^ *Kingella*,^20,21^ *Klebsiella*,^22^ *Moraxella*,^23,24^ *Morganella*,^25^ *Neisseria*,^26^ and *Proteus*^27^) and the G+ve genus *Enterococcus*.^28^ These designations were agreed by expert consensus between the microbiologists and ophthalmologists who are coauthors on this study. The classification of microorganisms as pathogenic was intentionally conservative to reduce the likelihood of false-positive identifications. Further data on the grouping of isolates from this study are presented in Supplemental Table, Supplemental Digital Content 1, http://links.lww.com/ICO/B916.

### Variables and Outcomes

The main outcome of the study was the proportion of recognized pathogen isolation. The analysis was conducted on the entire data set and on subgroups of patients with simultaneous contact lens, contact lens fluid/case, and/or corneal impression membranes.

### Ocular Sampling and Culturing

Samples were collected by ophthalmologists trained in proper sampling techniques. Routinely, 3 corneal impression membranes (CIM: PTFE Millicell Cell Culture Insert, 12 mm, pore size 0.4 μm; Merck Millipore, UK) from each patient's affected eye were collected, as described by Kaye et al.^29^ Before sample collection, preservative-free oxybuprocaine 0.4% was applied to the lower conjunctival fornix as a topical anesthetic. Using sterile gloves and avoiding contact with the eyelids or conjunctiva, a CIM was placed over the ulcer for 2 seconds. One CIM was immediately transferred to brain–heart infusion (BHI) broth for expansion and subsequent plating,^30^ while the other 2 were stored in sterile bijou tubes for molecular diagnosis. Whenever available, contact lenses (CL) and contact lens fluid/case (CLF) were also collected for culture and antimicrobial susceptibility testing.

Samples collected between January 2012 and October 2013 did not include CIMs but were instead collected via corneal scraping (CS) with a number 11 blade and placed in BHI.^29^ The BHI bottles were vortexed for 5 to 10 seconds, and 10 μL was inoculated onto blood, chocolate, and Sabouraud dextrose agar plates. A non-nutrient agar (Oxoid) seeded with *E. coli* was inoculated with 20 μL of broth for *Acanthamoeba* spp. culture. Blood and chocolate agar plates, along with the BHI broth, were incubated at 35 to 37°C, while Sabouraud plates were incubated at 30°C. Growth was checked after 24 and 48 hours, with Sabouraud plates monitored daily for 14 days. Isolates were identified using matrix-assisted laser desorption ionization-time-of-flight mass spectrometry throughout all the study period.

Cultures from contact lens cases were obtained by swabbing each chamber with a sterile swab and by directly inoculating the contact lens and contact lens fluid onto nutrient (blood agar and MacConkey agar) as well as non-nutrient agar. When contact lens fluid and case were not available, contact lenses were first put in BHI broth by the ophthalmologist collecting the sample and then inoculated after expansion in the broth.

### Statistical Analysis

Descriptive data were summarized using number (percentage), mean (SD), or median (interquartile range) where appropriate. Chi-square tests were used to compare the proportions of positive samples across all samples and subgroups. For positive samples, chi-square tests were used to compare the proportions of pathological/unknown significance isolates and the G+ve/G−ve ratio. Post hoc pairwise tests with Bonferroni correction for multiple comparisons were performed between different sample categories in case of significant chi-square tests.

Similarly, chi-square tests and post hoc pairwise tests were used to compare proportions of positive samples, pathogenic isolates, and G+ve/G−ve samples within sample type in the 4 different groups.

Logistic generalized linear mixed-models (GLMM) were used to estimate and compare the adjusted proportion of positive samples, the ratio of isolates of pathological versus unknown significance and the G+ve/G−ve ratio across groups. All models included a random intercept term specifying a unique patient identifier to account for intrasubject correlation of samples taken from the same patient. Tukey correction for multiple comparisons was applied to pairwise comparisons derived from the models across different sample types. A *P* value less than 0.05 was considered statistically significant. All analyses were conducted using R software version 4.2.2 (R Project for Statistical Computing, Vienna, Austria).

A summary of the data extraction, processing, and analysis workflow is visually presented in Figure 1.

**FIGURE 1.**
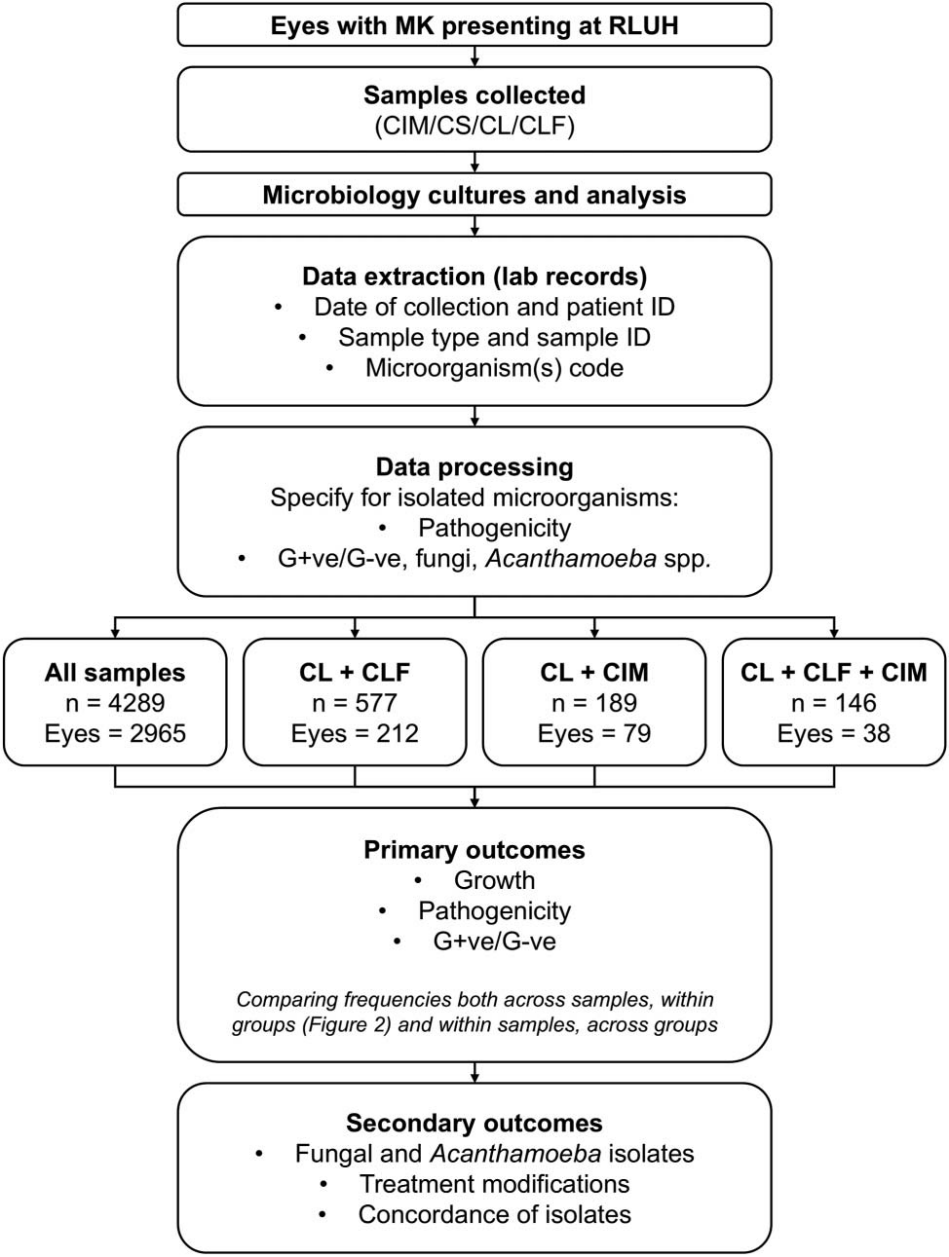
Visual summary of the data extraction, processing, and analysis workflow used in this study, illustrating the pathway from patient sampling through laboratory data retrieval, microbiological classification, and outcome analysis.

## RESULTS

In total, 4289 samples from 2965 patients were included. Most samples were obtained using a CIM (n = 2940, 68.5%), followed by CS (n = 566, 13.2%), CL (n = 466, 10.9%), and CLF (n = 317, 7.4%). A summary of the main outcomes from the study is presented in Table 1. There were 175 different types of micro-organisms isolated, of which 56 were considered as recognized pathogens. A list of all the isolates and their grouping (pathogen and unknown significance) is presented in Supplemental Table, Supplemental Digital Content 1, http://links.lww.com/ICO/B916**.** The proportions of positive isolates, pathogenic isolates, and G+ve/G−ve ratio estimated and compared from logistic GLMMs for all subgroups and sample types are presented in Figure 2.

**TABLE 1.** Distribution of Positive Samples, Pathogenic Isolates, and G+ve/G-ve Ratio Across Sample Types and Subgroups

	Sample Type	Positive Samples			Pathogenic Isolates			G+ve/G−ve Isolates		
All isolates	Contact lens	n = 4289	348/466	74.7%	n = 2919	269/531	50.7%	n = 2830	245/499	49.1%
	Contact lens fluid		23/317	7.3%		22/28	78.6%		6/11	54.5%
	Corneal impression membrane		1823/2940	62%		728/2218	32.8%		1860/2185	85.1%
	Corneal scrape		126/566	22.3%		71/142	50.0%		89/135	65.9%
										
CL + CLF	Contact lens	n = 577	228/299	76.3%	n = 373	178/351	50.7%	n = 332	139/324	42.9%
	Contact lens fluid		18/278	6.5%		18/22	81.8%		4/8	50.0%
										
CL + CIM	Contact lens	n = 189	80/100	80%	n = 193	71/118	60.2%	n = 186	53/113	46.9%
	Corneal impression membrane		54/89	60.7%		40/75	53.3%		53/73	72.6%
										
CL+CLF+CIM	Contact lens	n = 146	42/54	77.8%	n = 105	44/69	63.8%	n = 95	18/65	27.7%
	Contact lens fluid		7/51	13.7%		7/10	70.0%		4/5	80.0%
	Corneal impression membrane		20/41	48.8%		10/26	38.5%		17/25	68.0%

**FIGURE 2.**
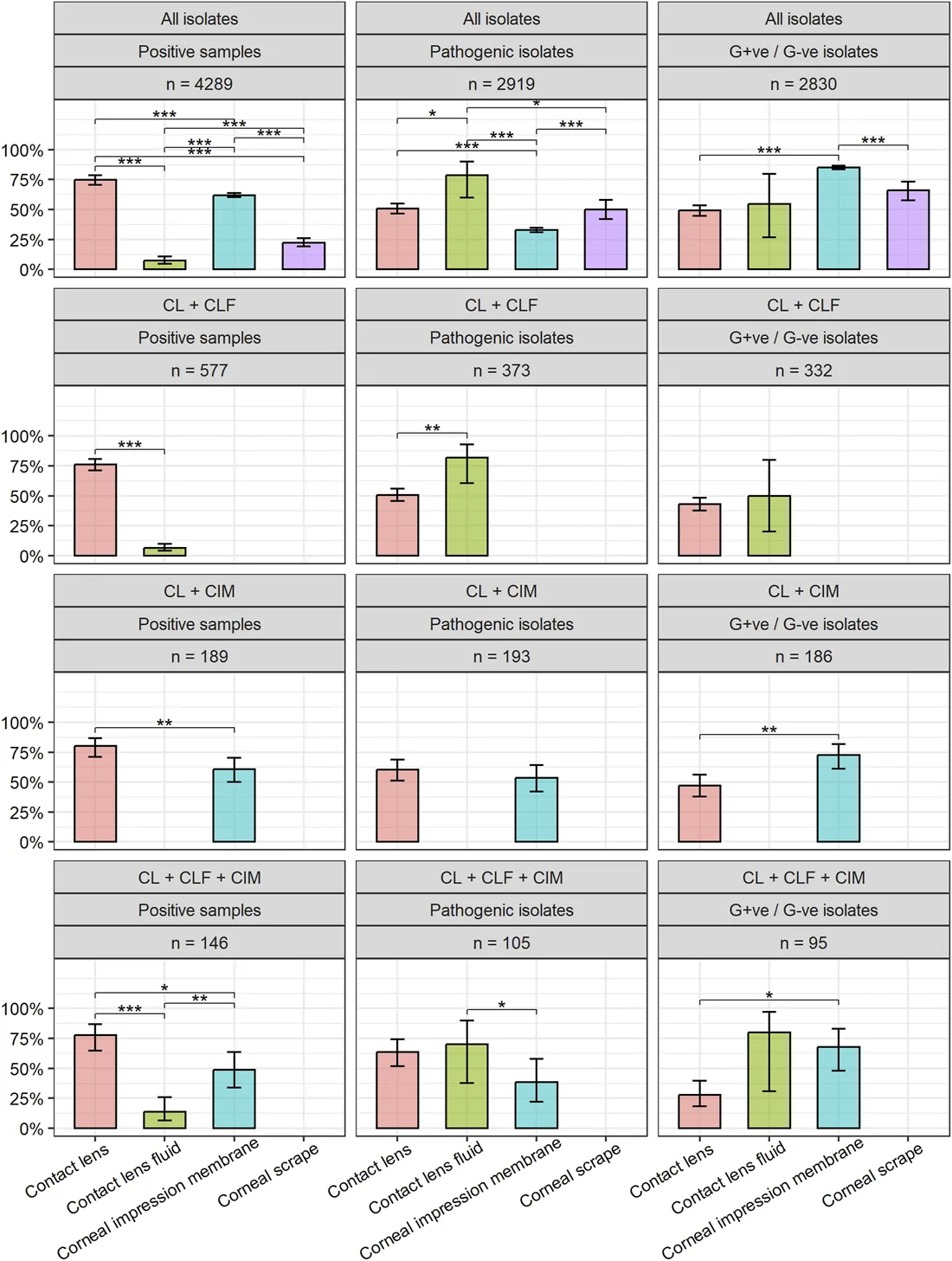
Bar plots showing the proportion of positive samples, pathogenic samples, and the ratio of Gram-positive and Gram-negative bacteria across different sample types. The different rows of the plot represent subgroups of samples collected simultaneously. Error bars represent the 95% confidence intervals of the estimated proportions. Pairwise *P* values are derived from generalized mixed effects models with Tukey correction for multiple comparisons. *P ≤ 0.05; **P ≤ 0.01; ***P ≤ 0.001.

### Isolation Rate

The isolation rate for all samples was 54.1% (n = 2319). CL cultures were positive in 74.7% of cases (348/466), followed by CIM ([1822/2940], 62.0%), CS ([126/566], 22.3%), and CLF ([23/317], 7.3%). All pairwise comparisons were significant (*P* <0.05).

Similar trends were observed in all subgroups. Among subjects who had both CL and CLF taken at the same time (n = 212), the isolation rate of the 577 total samples was 42.6% (n = 246). An average (SD) of 1.99 (1.2) CL and 1.3 (0.5) CLF samples was collected per subject in this subgroup. CLs were significantly more likely to yield an isolate than the CLF (76.3%, [228/299] vs. 6.5% [18/278], *P* <0.001).

Among subjects who had both CL and CIM taken at the same time (n = 79), the overall isolation rate of the 189 total samples was 70.9% (n = 134). An average (SD) of 1.7 (1.1) CL and 1.4 (0.9) CIM samples was collected per subject in this subgroup. CLs were more frequently positive than CIM (80.0% [80/100] vs. 60.7% [54/89], *P* = 0.006). In cases of negative CIM, CLs were positive in 65.7% of samples (23/35).

Finally, among subjects who had CL, CLF, and CIM taken at the same time (n = 38), the overall isolation rate of the 146 total samples was 47.2% (n = 69). An average (SD) of 2.1 (1.3) CL, 1.4 (0.6) CLF, and 1.2 (0.6) CIM samples was collected per subject in this subgroup. CLs were positive in 77.7% of cases (42/54), followed by CIM (48.8% [20/41]) and CLF (13.7% [7/51]). All pairwise comparisons were statistically significant (*P* <0.05).

Notably, CL, CLF, and CIM displayed no significant difference in proportion of isolates within sample type and across different patient subgroups (all isolates, CL vs. CLF, CL vs. CIM and CL vs. CLF vs. CIM). In cases of negative cultures for CIMs, CLs were positive in 85.7% of samples (24/28) and CLF was positive in 16.0% of samples (4/25).

### Samples from Which Pathogens Were Isolated

The 2319 positive samples in this study generated a total of 2919 individual isolates (mean [SD] number of isolates per sample, 1.14 [0.41]). CLs had on average the highest number of isolates per positive sample (mean [SD], 1.39 [0.66]), followed by CIM (1.14 [0.38]), CS (1.03 [0.24]), and CLF (1.02 [0.15]). Pathogens were identified in 1090 samples (47.0%). CLF had the highest overall proportion of pathogens (78.6% [22/28]), followed by CL (50.7% [269/531]), CS (50.0% [71/142]), and CIM (32.8% [728/2218]). No significant difference was found between the CL and CS groups. All other pairwise comparisons were significant. Among the paired CL and CLF samples (n = 577), the proportion of microorganisms isolated which were categorized as pathogens was 52.5% (196/373). CLF isolates were more frequently considered pathogens compared with CL (81.8% [18/22] vs. 50.7% [178/351], *P* = 0.007). Among subjects who had both CL and CIM taken at the same time (189 samples from 79 patients), the proportion of pathogens, among isolates, was 57.5% (111/193). There was no significant difference in the rate of isolation of pathogens between CL and CIM (60.2% [71/118] vs. 53.3% [40/75], *P* = 0.37). Of 47 isolates from 23 positive CL samples 61.7% (29/47) were pathogens. Finally, in the paired CL, CLF and CIM samples (146 samples from 38 patients), out of the 69 positive samples, the proportion of pathogens among isolates was 58.1% (61/105). CLF isolates were more frequently considered pathogens compared with paired CIM (70.0% [7/10] vs. 38.5% [10/26], *P* = 0.04). There was no significant difference with the proportion of pathogens in the CL group (63.8%, [44/69]). Of 24 positive CL samples and 4 positive CLF samples in case of negative CIMs, a total of 42 isolates were identified from CLs, with 66.6% of them being pathogens (28/42). All 4 isolates (100%) from positive CLF samples in cases with negative CIM grew Acanthamoeba.

### Gram Positive versus Gram-Negative Samples

Of the 2919 isolates in this study, most were G+ve isolates (n = 2200, 75.4%), followed by G-ve isolates (n = 630, 21.6%), fungi (n = 61, 2.1%), Acanthamoeba (n = 23, 0.8%), and mixed anaerobes (n = 5, 0.2%).

Among all bacterial isolates (n = 2830), the prevalence of G+ve isolates was significantly higher among CIM (85.1%, [1860/2185]) compared with CS (65.9% [89/135], *P* <0.001). There were no significant differences between CS and CLF (54.5%, [6/11], *P* = 0.66) and between CLF and CL (49.1% [245/499], *P* = 0.96). All other pairwise comparisons were significant (Table 1).

Among bacterial isolates from simultaneous CL and CLF samples (n = 332), there was no significant difference in the proportion of G+ve isolates (respectively, 42.9% [139/324] vs 50.0% [4/8], p = 0.73). Simultaneous CL and CIM generated 186 bacterial isolates, of which 106 were G+ve (57.0%). G+ve were more frequently detected with CIM than CL culture (72.6% [53/73] vs. 46.9% [53/113], p < 0.001). Finally, among 95 bacterial isolates generated from matched CL, CLF and CIM, 39 were G+ve (41.0%). G+ve isolates were found more frequently in CIM compared to CL (68.0% [17/25] vs. 27.7% [18/65], p = 0.003).

Estimates and 95% confidence intervals of the proportions of positive samples, pathogenic isolates, and G+ve/G-ve ratio for each subgroup with pairwise comparisons are presented in Supplemental Table, Supplemental Digital Content 2, http://links.lww.com/ICO/B917.

### Frequency Tests within Sample Types

Two proportions z-tests were also run within the same sample types (i.e., within CIM, CL, and CLF samples) to verify the consistency in the frequency of results across different groupings (i.e., across all isolates, CL vs. CLF, CL vs. CIM, and CL vs. CLF vs. CIM groups).

There were no significant differences in the proportion of positive samples in the same sample type for all sampling types across all isolates and in the CL versus CLF, CL versus CIM, and CL versus CLF versus CIM groups.

The proportion of pathogenic isolates was lower in CIM in the all-isolates group compared with the CL versus CIM group (32.8% [728/2218] vs. 53.3% [40/75], *P* = 0.001). All the other proportions of pathogenic isolates were comparable within the other samples. The proportion of G+ve isolates was higher in CL from the all-isolates group compared with the CL versus CLF versus CIM group (49.1% [245/499] vs. 27.7% [18/65], *P* = 0.01). Furthermore, the proportion of G+ve was higher in CIM from the all-isolates group compared with CL versus CIM (85.1% [1860/2185] vs. 72.6% [53/73], *P* = 0.02).

### Fungal and Acanthamoeba Isolates

Among all isolates (n = 2919), fungal growth was detected more frequently in the CLF group (14.3% [4/28]) than CIM (1.22% [27/2218], *P* <0.001) and CS (1.4% [2/142], *P* = 0.02). Detection of fungal growth was also significantly lower in CIM compared with CL (5.3% [28/531], *P* <0.001). Proportions of fungal growth were similar in the CL versus CLF, CL versus CIM and CL versus CLF versus CIM subgroups; however, no statistically significant difference could be detected for these subgroups (all *P* > 0.05).

Acanthamoeba growth was detected significantly more frequently among CLF isolates (46.4% [13/28]) compared with CIM (0.2% [5/2218], *P* <0.001) and CS (3.5% [5/142], *P* <0.001); of note, Acanthamoeba was never isolated in CL positive samples (0% [0/531]). Apart from samples originating from CLF, Acanthamoeba was also not detected in the subgroups of simultaneous CL versus CLF, CL versus CIM and CL versus CLF versus CIM, making pairwise comparisons not relevant.

### Treatment Modifications

Of 38 eyes for which all three samples were available (CIM, CL, and CLF), a total of eight eyes (21.1%) demonstrated concordance of cultures from the CIM with those of either CL or CLF. In 4 eyes (10.5%), the culturing of CL and/or CLF directly led to a change in treatment to adjust to culture results. Of note, five eyes showed clinical signs of Acanthamoeba keratitis and were treated with PHMB 0.06% monotherapy. Only 1 CLF sample, of the 38 paired samples, grew Acanthamoeba, but given no clinical features of amoebic keratitis, the patient did not receive antiamoebic treatment.

### Concordance of Isolates

Overall, among all positive samples from individual subjects, the agreement in the identification of the same microorganism was low to low-moderate. In particular, there was agreement between CIM and CS in 8/26 isolates (30.8%), between CIM and CL in 56/200 isolates (28%), between CL and CLF in 8/66 isolates (12.1%), and between CIM and CLF in 0/12 isolates (0%).

## DISCUSSION

In this study, we compared results of contact lens (CL) and contact lens fluid/case (CLF) with corneal samples (CIM and CS). We found that CL cultures had the highest positivity rate among sample types. Corneal samples obtained either using a scrape (CS) or membrane (CIM) were more likely to detect Gram-positive bacteria. Although CLF had a lower isolation rate, it provided the highest proportion of pathogens (e.g., Acanthamoeba and fungi). Importantly, CL and CLF cultures influenced treatment decisions in 10.5% of cases.

The high positivity rate of CL cultures in our study aligns with prior work demonstrating that microbial contamination of contact lenses is common and often exceeds that of corneal samples.^5,31^ This, however, is without distinction between recognized pathogens and nonpathogens. In particular, while CL cultures can provide additional microbiological data, their diagnostic value is challenged by potential contamination with environmental or commensal organisms.^5^ Concordance rates between corneal and CL cultures vary widely, reflecting a high variability in microbial overlap between these sample types.^6,11,12,32–34^ Although the number of microorganisms classified as pathogenic in this study represents a conservative estimate, if a distinction is made between recognized pathogens and nonpathogens, it becomes clear that CLF and CL are important for isolating microorganisms that may be responsible for the keratitis. In particular, we found that the number of positive CL samples in cases of negative CIMs ranged from 71% to 85%, and the percentage of pathogens in this group ranged from 61.7% to 66.6%.

Although previous studies did not distinguish between pathogens and microorganisms of unknown significance, Mela et al^31^ found that 67% of cases of CL-related MK had negative corneal scrapes, highlighting the added diagnostic benefit of culturing contact lenses and their storage solutions. Similarly, Das et al^11^ reported that 40% (20 of 50) of cases with negative corneal scrapings yielded positive results on contact lens cultures. In 113 patients with contact lens–related MK, Martins et al^33^ reported an 84% concordance between corneal scrapings and lens cultures for microorganism identification, with 100% concordance in fungal cases. Notably, Inoue et al^34^ reported a 30% microorganisms detection rate in fluid samples, with a concordance rate of 67%. Finally, Mukhtar et al^32^ reported a 7% concordance rate (9 of 135 cases) between corneal scrapings and contact lens cultures, with a significant number of cases showing negative corneal cultures. In all of these studies, however, no distinction was made between pathogen or a microorganism of unknown significance.

In our study, CL samples contained a higher proportion of G-ve bacteria. This finding is not surprising as most CL-related MK is associated with Gram-negative bacteria, particularly Gram-negative rods.^6^ The clinical relevance of organisms detected in CL cultures, however, remains a challenge, as contamination and colonization may not necessarily reflect the cause of the infection.^5^ CL cases or CLF in our study demonstrated a very low yield, likely due to the presence of antiseptics or disinfectants in lens solutions.^35^ Despite the lower overall positivity of CLF cultures, however, their ability to detect less common but recognized pathogens is important especially when protozoal or fungal infections are suspected. CLF cultures showed the highest detection rates for Acanthamoeba (46.4%) and fungi (14.3%), findings that align with existing literature demonstrating that lens storage cases and solutions might serve as reservoirs for these microorganisms.^36^ In support of this, previous work have reported detection rates of Acanthamoeba and/or fungal species in up to 24% in lens case cultures.^10^

This study has several strengths, including its large sample size and the comparison of multiple sample types spanning several years of microbiological data.

The laboratory data extraction process, however, cannot account for demographic and clinical data, which were extracted manually only for a subset of patients with simultaneous sampling. Furthermore, the retrospective nature of the study introduces potential selection bias, as patients could have undergone antibiotic eye drops treatment before sampling which could affect the isolation rate, not all patients had all types of sample types collected, and CL-related materials were only available for testing in a subset of cases. This is consistent with previous research highlighting the low availability of lens cases and solutions for examination, suggesting a gap in patient awareness regarding the importance of bringing these materials when seeking medical care. This is augmented by the popularity of daily disposable CLs. Nonetheless, encouraging patients to provide their CL and CLF samples increases the likelihood of detecting a pathogen particularly in cases where corneal cultures are negative. Although this approach may involve additional laboratory time and associated costs, the diagnostic and clinical utility of improved pathogen detection may justify the resources required.

In conclusion, our results support the process of sampling CLs and cases in patients with CL-associated MK to improve the detection rate of recognized pathogens, possibly guiding specific treatment.

## Supplementary Material
