# Supplementary material for: Diagnostic Yield of Corneal, Contact Lens, and Lens Fluid Cultures in Microbial Keratitis: A 12-Year Single-Center Study
Source: Cornea. 2025 Dec 11;45(11):1401–7. doi: 10.1097/ICO.0000000000004045 (PMC13626590; doi:10.1097/ICO.0000000000004045)
Supplement: Supplementary file 1 [file cornea-45-1401-s001.docx]

|  | **Pathogens** |  |  |  |  |  | **Unknown significance** |  |  |  |  |
| --- | --- | --- | --- | --- | --- | --- | --- | --- | --- | --- | --- |
| **Stain** | **Organism** | **CL** | **CLF** | **CIM** | **CS** | **Stain** | **Organism** | **CL** | **CLF** | **CIM** | **CS** |
| Acanthamoeba | *Acanthamoeba sp.* | - | 13 | 5 | 5 | Fungi | *Chaetomium sp.* | - | - | 1 | - |
| Fungi | *Aspergillus flavus* | - | - | 1 | - | Fungi | *Cryptococcus sp.* | - | - | 1 | - |
| Fungi | *Aspergillus niger* | - | - | 1 | 1 | Fungi | *Fungus* | - | 2 | 4 | 1 |
| Fungi | *Candida albicans* | - | - | 4 | - | Fungi | *Paecilomyces variotii* | - | - | 1 | - |
| Fungi | *Candida dubliniensis* | - | - | 1 | - | Fungi | *Penicillium sp.* | - | - | 5 | - |
| Fungi | *Candida guilliermondii* | 7 | 2 | - | - | Fungi | *Purpureocillium lilacinum* | - | - | 1 | - |
| Fungi | *Candida lusitaniae* | 1 | - | - | - | Fungi | *Ulocladium chartarum* | - | - | 1 | - |
| Fungi | *Candida parapsilosis* | 17 | - | 2 | - | Fungi | *Yeasts* | 1 | - | 2 | - |
| Fungi | *Candida pararugosa* | - | - | 1 | - | Gram-positive | *Aerococcus viridans* | - | - | 1 | - |
| Fungi | *Fusarium sp.* | 2 | - | 1 | - | Gram-positive | *Alpha haemolytic streptococcus* | 16 | - | 44 | 5 |
| Gram-positive | *Enterococcus avium* | - | - | 1 | - | Gram-positive | *Alpha haemolytic streptococcus (2)* | - | - | 2 | 1 |
| Gram-positive | *Enterococcus durans* | - | - | 1 | - | Gram-positive | *Anaerobic Gram Positive Coccus* | - | - | - | 1 |
| Gram-positive | *Enterococcus faecalis* | 6 | - | 19 | - | Gram-positive | *Bacillus cereus* | 4 | - | 1 | - |
| Gram-positive | *Enterococcus faecium* | - | - | 1 | - | Gram-positive | *Bacillus licheniformis* | - | - | 1 | - |
| Gram-positive | *Enterococcus sp.* | 3 | - | 1 | - | Gram-positive | *Bacillus mycoides* | 1 | - | - | - |
| Gram-positive | *Group A Beta haemolytic streptococcus* | - | - | 3 | - | Gram-positive | *Bacillus pumilis* | - | - | 1 | - |
| Gram-positive | *Staphylococcus aureus* | 33 | 2 | 336 | 17 | Gram-positive | *Bacillus sp.* | 12 | 1 | 19 | 1 |
| Gram-positive | *Staphylococcus aureus (MRSA)* | 2 | - | 10 | - | Gram-positive | *Bacillus subtilis* | - | - | 1 | - |
| Gram-positive | *Streptococcus pneumoniae* | 3 | - | 48 | 9 | Gram-positive | *Bacillus thuringiensis* | 1 | - | - | - |
| Gram-positive | *Vancomycin Resistant Enterococci* | - | - | 2 | - | Gram-positive | *Brevibacterium casei* | - | - | 1 | - |
| Gram-negative | *Citrobacter freundii* | 1 | - | - | 1 | Gram-positive | *Clostridium perfringens* | 2 | - | - | - |
| Gram-negative | *Citrobacter koseri* | - | - | 1 | - | Gram-positive | *Clostridium sp.* | 2 | - | - | - |
| Gram-negative | *Enterobacter aerogenes* | - | - | 1 | - | Gram-positive | *Coagulase negative staphylococcus* | 34 | - | 73 | 34 |
| Gram-negative | *Enterobacter cloacae* | 5 | 1 | 1 | - | Gram-positive | *Coagulase negative staphylococcus (2)* | 7 | - | 1 | - |
| Gram-negative | *Enterobacter sp.* | 11 | - | 1 | 1 | Gram-positive | *Corynebacterium accolens* | - | - | 3 | - |
| Gram-negative | *Escherichia coli* | 1 | - | 33 | 5 | Gram-positive | *Corynebacterium amycolatum* | 2 | - | 1 | - |
| Gram-negative | *Escherichia coli (CARB+)* | - | - | 3 | - | Gram-positive | *Corynebacterium aurimucosum* | - | - | 2 | - |
| Gram-negative | *Escherichia vulneris* | - | - | 1 | - | Gram-positive | *Corynebacterium bovis* | - | - | 3 | - |
| Gram-negative | *Haemophilus influenzae* | - | - | 3 | 2 | Gram-positive | *Corynebacterium glucuronolyticum* | - | - | 1 | - |
| Gram-negative | *Haemophilus parainfluenzae* | - | - | 1 | 1 | Gram-positive | *Corynebacterium macginleyi* | 1 | - | 2 | - |
| Gram-negative | *Kingella kingae* | - | - | - | 1 | Gram-positive | *Corynebacterium propinquum* | - | - | 7 | 1 |
| Gram-negative | *Klebsiella oxytoca* | 25 | 1 | 6 | 1 | Gram-positive | *Corynebacterium pseudodiphtheriticum* | 2 | - | 2 | 1 |
| Gram-negative | *Klebsiella pneumoniae* | 7 | - | 4 | - | Gram-positive | *Corynebacterium sp.* | - | - | 9 | - |
| Gram-negative | *Klebsiella sp* | 4 | - | 2 | - | Gram-positive | *Corynebacterium striatum* | - | - | 9 | - |
| Gram-negative | *Klebsiella variicola* | 1 | - | - | - | Gram-positive | *Diphtheroids* | 1 | - | 6 | - |
| Gram-negative | *Moraxella catarrhalis* | - | - | 4 | 1 | Gram-positive | *Gemella morbillorum* | - | - | 1 | - |
| Gram-negative | *Moraxella lacunata* | - | - | 4 | 2 | Gram-positive | *Gram positive bacilli* | - | - | 1 | 1 |
| Gram-negative | *Moraxella nonliquefaciens* | - | - | 13 | 6 | Gram-positive | *Group B Beta haemolytic streptococcus* | 2 | - | 13 | - |
| Gram-negative | *Moraxella osloensis* | 1 | - | 5 | 1 | Gram-positive | *Group C Beta haemolytic streptococcus* | 2 | - | 4 | - |
| Gram-negative | *Moraxella sp.* | - | - | 18 | - | Gram-positive | *Group G Beta haemolytic streptococcus* | - | - | 5 | 1 |
| Gram-negative | *Morganella morganii* | 2 | - | 5 | - | Gram-positive | *Kocuria palustris* | - | - | 1 | - |
| Gram-negative | *Neisseria meningitidis* | - | - | 1 | 1 | Gram-positive | *Kocuria rhizophila* | - | - | 10 | - |
| Gram-negative | *Neisseria sp.* | - | - | - | 1 | Gram-positive | *Lactobacillus sp.* | - | - | 1 | - |
| Gram-negative | *Proteus mirabilis* | 6 | - | 21 | 1 | Gram-positive | *Microbacterium sp.* | - | 1 | 1 | - |
| Gram-negative | *Proteus sp.* | - | - | 1 | 2 | Gram-positive | *Micrococcus luteus* | 1 | - | 30 | 1 |
| Gram-negative | *Proteus vulgaris* | - | - | 1 | - | Gram-positive | *Micrococcus sp.* | 2 | - | 4 | - |
| Gram-negative | *Pseudomonas aeruginosa* | 53 | 2 | 101 | 8 | Gram-positive | *Mixed alpha-haemolytic streptococci* | - | - | 1 | - |
| Gram-negative | *Serratia grimesii* | - | - | 1 | 1 | Gram-positive | *Mixed coagulase negative staphylococci* | 22 | 1 | 316 | - |
| Gram-negative | *Serratia liquefaciens* | 24 | 1 | 13 | 2 | Gram-positive | *Non haemolytic streptococcus* | 1 | - | - | 1 |
| Gram-negative | *Serratia marcescens* | 39 | - | 42 | 1 | Gram-positive | *Peribacillus simplex* | - | - | 2 | - |
| Gram-negative | *Serratia plymuthica* | 1 | - | - | - | Gram-positive | *Rothia mucilaginosa* | - | - | 1 | - |
| Gram-negative | *Serratia sp #2* | 1 | - | - | - | Gram-positive | *Rothia sp.* | - | - | 2 | - |
| Gram-negative | *Serratia sp.* | 13 | - | 3 | - | Gram-positive | *Staphylococcus capitis* | 6 | - | 64 | 1 |
|  |  |  |  |  |  | Gram-positive | *Staphylococcus caprae* | 1 | - | 3 | - |
|  |  |  |  |  |  | Gram-positive | *Staphylococcus epidermidis* | 37 | - | 563 | 11 |
|  |  |  |  |  |  | Gram-positive | *Staphylococcus epidermidis (2)* | 1 | - | 13 | - |
|  |  |  |  |  |  | Gram-positive | *Staphylococcus equorum* | - | - | 1 | - |
|  |  |  |  |  |  | Gram-positive | *Staphylococcus haemolyticus* | 1 | - | 13 | - |
|  |  |  |  |  |  | Gram-positive | *Staphylococcus hominis* | 4 | - | 32 | - |
|  |  |  |  |  |  | Gram-positive | *Staphylococcus lugdunensis* | - | - | 13 | - |
|  |  |  |  |  |  | Gram-positive | *Staphylococcus pasteuri* | 6 | - | 8 | - |
|  |  |  |  |  |  | Gram-positive | *Staphylococcus pettenkoferi* | - | - | 1 | - |
|  |  |  |  |  |  | Gram-positive | *Staphylococcus saprophyticus* | - | - | 6 | - |
|  |  |  |  |  |  | Gram-positive | *Staphylococcus simulans* | 1 | - | - | - |
|  |  |  |  |  |  | Gram-positive | *Staphylococcus sp.* | - | - | 2 | - |
|  |  |  |  |  |  | Gram-positive | *Staphylococcus succinus* | - | - | 1 | - |
|  |  |  |  |  |  | Gram-positive | *Staphylococcus warneri* | 8 | 1 | 28 | 2 |
|  |  |  |  |  |  | Gram-positive | *Staphylococcus xylosus* | - | - | 1 | - |
|  |  |  |  |  |  | Gram-positive | *Streptococcus cristatus* | - | - | 2 | - |
|  |  |  |  |  |  | Gram-positive | *Streptococcus gordonii* | - | - | 2 | - |
|  |  |  |  |  |  | Gram-positive | *Streptococcus milleri* | 2 | - | 6 | 1 |
|  |  |  |  |  |  | Gram-positive | *Streptococcus mitis* | 6 | - | 20 | - |
|  |  |  |  |  |  | Gram-positive | *Streptococcus oralis* | 8 | - | 32 | - |
|  |  |  |  |  |  | Gram-positive | *Streptococcus parasanguinis* | 1 | - | 16 | - |
|  |  |  |  |  |  | Gram-positive | *Streptococcus parasanguis* | - | - | 6 | - |
|  |  |  |  |  |  | Gram-positive | *Streptococcus salivarius* | 1 | - | 11 | - |
|  |  |  |  |  |  | Gram-positive | *Streptococcus sanguinis* | - | - | 5 | - |
|  |  |  |  |  |  | Gram-positive | *Streptococcus sanguis* | - | - | 2 | - |
|  |  |  |  |  |  | Gram-positive | *Streptococcus vestibularis* | - | - | 4 | - |
|  |  |  |  |  |  | Gram-negative | *Achromobacter sp.* | 4 | - | - | - |
|  |  |  |  |  |  | Gram-negative | *Achromobacter xylosoxidans* | 4 | - | - | - |
|  |  |  |  |  |  | Gram-negative | *Acinetobacter baumannii* | 1 | - | 2 | - |
|  |  |  |  |  |  | Gram-negative | *Acinetobacter lwoffii* | - | - | 3 | 1 |
|  |  |  |  |  |  | Gram-negative | *Acinetobacter pittii* | - | - | 1 | - |
|  |  |  |  |  |  | Gram-negative | *Acinetobacter radioresistens* | - | - | 1 | - |
|  |  |  |  |  |  | Gram-negative | *Acinetobacter sp* | 1 | - | 4 | - |
|  |  |  |  |  |  | Gram-negative | *Acinetobacter ursingii* | 2 | - | 1 | - |
|  |  |  |  |  |  | Gram-negative | *Aeromonas hydrophila* | 4 | - | - | - |
|  |  |  |  |  |  | Gram-negative | *Alcaligenes faecalis* | 2 | - | 2 | - |
|  |  |  |  |  |  | Gram-negative | *Burkholderia cepacia* | 1 | - | - | - |
|  |  |  |  |  |  | Gram-negative | *Chryseobacterium sp.* | 2 | - | - | - |
|  |  |  |  |  |  | Gram-negative | *Coliform sp.* | 2 | - | 1 | - |
|  |  |  |  |  |  | Gram-negative | *Delftia acidovorans* | 2 | - | 1 | 1 |
|  |  |  |  |  |  | Gram-negative | *Delftia sp* | 1 | - | - | - |
|  |  |  |  |  |  | Gram-negative | *Flavobacterium sp.* | 1 | - | - | 1 |
|  |  |  |  |  |  | Gram-negative | *Gram negative bacilli* | - | - | - | 1 |
|  |  |  |  |  |  | Gram-negative | *Massilia timonae* | - | - | - | 1 |
|  |  |  |  |  |  | Gram-negative | *Mixed coliforms* | 6 | - | - | - |
|  |  |  |  |  |  | Gram-negative | *Non-lactose fermenting coliform* | 1 | - | - | 1 |
|  |  |  |  |  |  | Gram-negative | *Paenibacillus sp.* | - | - | 2 | 1 |
|  |  |  |  |  |  | Gram-negative | *Pandoraea sp* | 2 | - | - | - |
|  |  |  |  |  |  | Gram-negative | *Pantoea agglomerans* | - | - | 1 | - |
|  |  |  |  |  |  | Gram-negative | *Pantoea septica* | - | - | 1 | - |
|  |  |  |  |  |  | Gram-negative | *Pseudomonas fluorescens* | - | - | 1 | - |
|  |  |  |  |  |  | Gram-negative | *Pseudomonas luteola* | 1 | - | - | - |
|  |  |  |  |  |  | Gram-negative | *Pseudomonas putida* | 1 | - | - | - |
|  |  |  |  |  |  | Gram-negative | *Pseudomonas sp.* | 6 | - | 7 | - |
|  |  |  |  |  |  | Gram-negative | *Raoultella ornithinolytica* | 3 | - | - | - |
|  |  |  |  |  |  | Gram-negative | *Raoultella planticola* | - | - | 1 | - |
|  |  |  |  |  |  | Gram-negative | *Raoultella sp* | 3 | - | - | - |
|  |  |  |  |  |  | Gram-negative | *Roseomonas mucosa* | - | - | 1 | - |
|  |  |  |  |  |  | Gram-negative | *Stenotrophomonas maltophilia* | 9 | - | 4 | - |
|  |  |  |  |  |  | Gram-negative | *Veillonella parvula* | - | - | 1 | - |
|  |  |  |  |  |  | Unknown | *Mixed anaerobes* | 4 | - | 1 | - |
