# Supplementary material for: Diagnostic Yield of Corneal, Contact Lens, and Lens Fluid Cultures in Microbial Keratitis: A 12-Year Single-Center Study
Source: Cornea. 2025 Dec 11;45(11):1401–7. doi: 10.1097/ICO.0000000000004045 (PMC13626590; doi:10.1097/ICO.0000000000004045)
Supplement: Supplementary file 2 [file cornea-45-1401-s002.docx]

**Supplementary Table 2**. Estimated proportions and pairwise comparisons across sample types and subgroups.

|  |  |  | **Positive samples** | | | |  | **Pathogenic isolates** | | | |  | **G+ve / G-ve isolates** | | | |
| --- | --- | --- | --- | --- | --- | --- | --- | --- | --- | --- | --- | --- | --- | --- | --- | --- |
|  |  |  | **Estimate (95% CI)** | **Pairwise comparisons** | | |  | **Estimate (95% CI)** | **Pairwise comparisons** | | |  | **Estimate (95% CI)** | **Pairwise comparisons** | | |
|  |  |  |  | ***CLF*** | ***CIM*** | ***CS*** |  |  | ***CLF*** | ***CIM*** | ***CS*** |  |  | ***CLF*** | ***CIM*** | ***CS*** |
|  |  |  |  |  |  |  |  |  |  |  |  |  |  |  |  |  |
| All isolates | *CL* |  | 74.7% (70.5%, 78.4%) | <0.001 | <0.001 | <0.001 |  | 50.7% (46.4%, 54.9%) | 0.01 | <0.001 | 1 |  | 49.1% (44.7%, 53.5%) | 0.98 | <0.001 | 0.16 |
|  | *CLF* |  | 7.3% (4.9%, 10.7%) | - | <0.001 | <0.001 |  | 78.6% (59.8%, 90%) | - | <0.001 | 0.02 |  | 54.5% (26.8%, 79.7%) | - | 0.2 | 0.99 |
|  | *CIM* |  | 62% (60.2%, 63.7%) | - | - | <0.001 |  | 32.8% (30.9%, 34.8%) | - | - | <0.001 |  | 85.1% (83.6%, 86.6%) | - | - | <0.001 |
|  | *CS* |  | 22.3% (19%, 25.9%) | - | - | - |  | 50% (41.8%, 58.2%) | - | - | - |  | 65.9% (57.5%, 73.4%) | - | - | - |
|  |  |  |  |  |  |  |  |  |  |  |  |  |  |  |  |  |
| CL + CLF | *CL* |  | 76.3% (71.1%, 80.7%) | <0.001 | - | - |  | 50.7% (45.5%, 55.9%) | 0.004 | - | - |  | 42.9% (37.6%, 48.4%) | 0.47 | - | - |
|  | *CLF* |  | 6.5% (4.1%, 10%) | - | - | - |  | 81.8% (60.4%, 93%) | - | - | - |  | 50% (20%, 80%) | - | - | - |
|  |  |  |  |  |  |  |  |  |  |  |  |  |  |  |  |  |
| CL + CIM | *CL* |  | 80% (71%, 86.7%) | - | 0.002 | - |  | 60.2% (51.1%, 68.6%) | - | 0.28 | - |  | 46.9% (37.9%, 56.1%) | - | 0.01 | - |
|  | *CIM* |  | 60.7% (50.2%, 70.2%) | - | - | - |  | 53.3% (42.1%, 64.3%) | - | - | - |  | 72.6% (61.3%, 81.6%) | - | - | - |
|  |  |  |  |  |  |  |  |  |  |  |  |  |  |  |  |  |
| CL + CLF + CIM | *CL* |  | 77.8% (64.8%, 86.9%) | <0.001 | 0.01 | - |  | 63.8% (51.9%, 74.2%) | 0.29 | 0.07 | - |  | 27.7% (18.2%, 39.7%) | 0.33 | 0.01 | - |
|  | *CLF* |  | 13.7% (6.7%, 26.1%) | - | 0.002 | - |  | 70% (37.6%, 90%) | - | 0.04 | - |  | 80% (30.9%, 97.3%) | - | 0.89 | - |
|  | *CIM* |  | 48.8% (34%, 63.7%) | - | - | - |  | 38.5% (22.1%, 57.9%) | - | - | - |  | 68% (47.8%, 83.1%) | - | - | - |
|  |  |  |  |  |  |  |  |  |  |  |  |  |  |  |  |  |
